# Estimating the Time Since Deposition of Saliva Stains With a Targeted Bacterial DNA Approach: A Proof-of-Principle Study

**DOI:** 10.3389/fmicb.2021.647933

**Published:** 2021-06-02

**Authors:** Celia Díez López, Manfred Kayser, Athina Vidaki

**Affiliations:** Department of Genetic Identification, Erasmus MC University Medical Center Rotterdam, Rotterdam, Netherlands

**Keywords:** forensic genetics, microbial forensics, stain deposition time, prediction, qPCR, bacterial DNA, saliva stains

## Abstract

Information on the time when a stain was deposited at a crime scene can be valuable in forensic investigations. It can link a DNA-identified stain donor with a crime or provide a post-mortem interval estimation in cases with cadavers. The available methods for estimating stain deposition time have limitations of different types and magnitudes. In this proof-of-principle study we investigated for the first time the use of microbial DNA for this purpose in human saliva stains. First, we identified the most abundant and frequent bacterial species in saliva using publicly available 16S rRNA gene next generation sequencing (NGS) data from 1,848 samples. Next, we assessed time-dependent changes in 15 identified species using de-novo 16S rRNA gene NGS in the saliva stains of two individuals exposed to indoor conditions for up to 1 year. We selected four bacterial species, i.e., *Fusobacterium periodonticum, Haemophilus parainfluenzae, Veillonella dispar*, and *Veillonella parvula* showing significant time-dependent changes and developed a 4-plex qPCR assay for their targeted analysis. Then, we analyzed the saliva stains of 15 individuals exposed to indoor conditions for up to 1 month. Bacterial counts generally increased with time and explained 54.9% of the variation (*p* = <2.2E–16). Time since deposition explained ≥86.5% and ≥88.9% of the variation in each individual and species, respectively (*p* = <2.2E–16). Finally, based on sample duplicates we built and tested multiple linear regression models for predicting the stain deposition time at an individual level, resulting in an average mean absolute error (MAE) of 5 days (ranging 3.3–7.8 days). Overall, the deposition time of 181 (81.5%) stains was correctly predicted within 1 week. Prediction models were also assessed in stains exposed to similar conditions up to 1 month 7 months later, resulting in an average MAE of 8.8 days (ranging 3.9–16.9 days). Our proof-of-principle study suggests the potential of the DNA profiling of human commensal bacteria as a method of estimating saliva stains time since deposition in the forensic scenario, which may be expanded to other forensically relevant tissues. The study considers practical applications of this novel approach, but various forensic developmental validation and implementation criteria will need to be met in more dedicated studies in the future.

## Introduction

In routine forensic investigations, DNA profiling based on short tandem repeats (STRs) is the gold standard for identifying the individuals who left a biological sample at the crime scene (Butler, 2004). However, the presence of a person’s DNA at a crime scene does not necessarily allow us to conclude that the sample donor is the perpetrator, which is typically done in court using additional (non-genetic) information. One important additional piece of information, which can be crucial to solving a case, is knowledge of the time frame when the person identified by DNA-based profiling left the biological stain at the scene. Knowing the time since deposition of a crime scene stain can help the police assess the alibis of suspects or provide investigative information to search for the right suspect. Moreover, when multiple biological traces belonging to different donors are found at a scene, information on their time since deposition may help investigators select the ones with the highest investigative value for further analysis, in cases where the time of the crime is known. Furthermore, in some missing person cases, such knowledge of the time of stain deposition on relevant items (such as clothing) might also be linked with the time a person went missing if it is unknown. In crime scenes involving (parts of) a corpse, estimation of the time since deposition of the stains found around/on the body can serve as an additional method for determining the time since death, i.e., post-mortem interval (PMI).

For estimating the time since deposition of human biological stains, the most studied molecular approach to date is the differential time-dependent degradation of human RNA, using mainly mRNA markers (Bauer et al., 2003; Anderson et al., 2011; Weinbrecht et al., 2017; Amany et al., 2018; Fu and Allen, 2019; Asaghiar and Williams, 2020), but also miRNA markers (Amany et al., 2018; Alshehhi and Haddrill, 2019). In principle, RNA decay continues *ex vivo* after a stain has been deposited, even if the biological material is dehydrated (Bauer, 2007). However, not all markers investigated for their potential time-dependent degradation are informative, as outlined in published studies, due to their reported time-stability for months (Watanabe et al., 2017; Amany et al., 2018; Alshehhi and Haddrill, 2019) or even a year (Alshehhi and Haddrill, 2019). Nevertheless, the majority of studies to date have reported the time-dependent decay of selected RNA markers, and a few have also attempted to use this for estimating the time since deposition of body fluid stains.

A qPCR-based study on degradation profiles of two human hypoxia sensitive mRNA markers up to a month obtained mean absolute error (MAE) values of 2.7, 3.5, and 6.4 days in blood, saliva, and semen stains, respectively (Asaghiar and Williams, 2020). However, the sample size was small (*n* = 5 for each body fluid) and stain exposure did not mimic realistic forensic scenarios. In the cases of saliva and semen, fluids were left in tubes until swabbed rather than left to dry as stains. Bauer et al. (2003) analyzed two mRNA markers in dried bloodstains using qPCR under the hypothesis that the 5′-end degrades at a faster rate than the 3′-end in mRNA, and that relative degradation could be used to estimate the time since deposition of the stains. Significant levels in mRNA degradation were only reported in stains with large deposition time differences of at least 4–5 years, resulting in very large estimation intervals of several months or even a few years. Another qPCR-based study analyzed four mRNA markers in dried bloodstains under the same previous hypothesis and reported a time estimation error of 2–4 weeks for stains exposed less than 6 months and 4–6 weeks for stains exposed between six and 12 months (Fu and Allen, 2019). Furthermore (Alshehhi and Haddrill, 2019) employed qPCR targeting two and four mRNA/miRNA markers in dried saliva and semen stains, respectively, for up to a year. On the one hand, the mRNA markers showed large fluctuation, no degradation, or were not detected at all due to the assay’s sensitivity past 90 days. On the other hand, the miRNA markers remained stable across all analyzed time points, making them not suitable for investigating time-dependent changes. Lastly, another study employed RNA next-generation sequencing (NGS) to analyze the potential time-dependency of the transcripts present in dried blood, saliva, semen, and vaginal fluid stains for up to a year (Weinbrecht et al., 2017). Particularly for the saliva transcripts, abundance values decreased rapidly and erratically; hence, no comprehensive analysis could be performed. For other stains, the time-dependency of transcripts was useful for a limited time period of less than a year. Overall, a RNA-based approach for estimating the time since deposition of stains could be promising but suffers from significant drawbacks including inter-individual degradation variation.

The human microbiome has been recently proposed as a promising tool in forensic science, especially for forensically relevant topics, for which other approaches present challenges and limitations. For example, the human microbiome has proven to be a promising forensic tool for PMI estimation based on predictable succession and colonization of microorganisms over time at different body sites (Hyde et al., 2013; Adserias-Garriga et al., 2017; Pechal et al., 2018; Dash and Das, 2020). However, caution must be taken due to the environmental (Pechal et al., 2017; Dash and Das, 2020) and individual-specific (Dash and Das, 2020) factors affecting time-dependent changes. The human microbiome, particularly the skin microbiome, can also serve as a kind of “fingerprint” that is transferred to touched objects, which is promising for individual identification in cases where recovered human DNA is not sufficient for obtaining a STR profile (“touched” samples; Schmedes et al., 2017, 2018; Yang et al., 2019). Additionally, we also showed that the human microbiome is suitable for the identification of the body site of origin of human body fluid stains, which can be of great value in crime scene reconstruction. For instance, when it comes to crime scene stains that contain epithelial cells from different body sites of origin including skin, saliva, and vaginal fluids (Díez López et al., 2019), and bloodstains from different body sites of origin including venous/arterial blood, menstrual blood, nasal blood, or blood from skin injuries (Díez López et al., 2020), where previous molecular approaches such as RNA-based ones have limitations (Haas et al., 2014; Holtkotter et al., 2017; Ingold et al., 2018). On top of these previously investigated forensic microbiome applications, we envisioned it a promising tool for estimating the time since deposition of human biological stains at a crime scene, which has not been studied yet.

Particularly, the oral human microbiome has been extensively characterized (Escapa et al., 2018) and the microorganisms living in the oral cavity comprise the second largest and most diverse microbial community of the human body (Huttenhower et al., 2012) after the gut. Notably, 1 mL of saliva in healthy adults is estimated to contain approximately 100 million bacterial cells (Curtis et al., 2011). Considering the normal salivary flow rate to be around 750 mL/day, 8 × 10^10^ bacterial cells are shed daily from the oral surfaces (Curtis et al., 2011). As a result, human saliva samples are likely to contain a high number of bacterial cells, including dried saliva stains found at crime scenes that often are small and based on just a few microliters (μL) of liquid saliva. Additionally, it has been shown that the “core” oral microbiome, which can be defined as the taxa shared among unrelated individuals (Zaura et al., 2009), is quite large, and is larger than in other body sites such as the gut or skin (Costello et al., 2009). Finally, the oral microbiome has shown a high degree of *in vivo* time-wise stability within an individual, with no significant changes over months (Costello et al., 2009; Lazarevic et al., 2010; Zhu et al., 2012) and even a few years (Stahringer et al., 2012). The time-stable biological information of any biomarker used in forensics is crucial in investigations, especially in approaches where old crime scene samples may be used retrospectively, such as for estimating the time since stain deposition. To date, only a few studies have investigated the time-dependent changes in the microbiome in dried saliva samples exposed to indoor conditions (Dobay et al., 2019; Salzmann et al., 2019), and due to their small sample size, no meaningful conclusions could be made on how suitable the approach is for forensic purposes.

This proof-of-principle study is the first to investigate the potential of the genetic profiling of human saliva commensal bacteria for estimating the time since deposition of saliva stains, which has promising applications in future forensic scenarios. We first identified the most abundant and most frequent bacterial species in saliva from a large publicly available 16S rRNA gene NGS data set. Next, we assessed time-dependent changes in the relative abundance of the top identified bacterial target species in 16S rRNA gene NGS data produced from dried saliva stains exposed to indoor long-term conditions for up to 1 year. Based on the observed significant time-dependent changes, we further selected four bacterial species, for which we developed a multiplex qPCR assay. Finally, this assay was used to analyze dried saliva stains exposed to indoor short-term conditions with various sample storage times up to 1 month.

## Materials and Methods

### Saliva Microbiome Data Sets

Publicly available human saliva 16S rRNA gene NGS data from two previously published studies were obtained from the European Bioinformatics Institute (EMBL-EBI). These studies included data from three cohorts: the American Cancer Society Cancer Prevention Study II (ACS CPS-II; *N* = 543; Wu et al., 2016), and the Prostate, Lung, Colorectal, and Ovarian (PLCO) Cancer Screening Trial (*N* = 661; Wu et al., 2016) from which the produced microbiome data was published as part of the same study; and the American Gut Project (AGP; *N* = 1,089; McDonald et al., 2018). The accession numbers were PRJNA434300, PRJNA434312, and PRJEB11419, respectively.

The metadata of the studies were accessed via the National Center for Biotechnology Information (NCBI) and matched to the corresponding sample identifiers using custom Python scripts to create flat metadata tables. In the first study (ACS CPS-II/PLCO), quality control sample replicates were removed to avoid data redundancy. We also discarded samples with missing metadata information for age, sex, and/or ethnicity. We also removed samples obtained from donors less than 15 years old, given that differences between adult and youth saliva microbiomes are expected (Burcham et al., 2020).

### Most Abundant and Frequent Bacterial Species in Saliva

**FIGURE 1:**
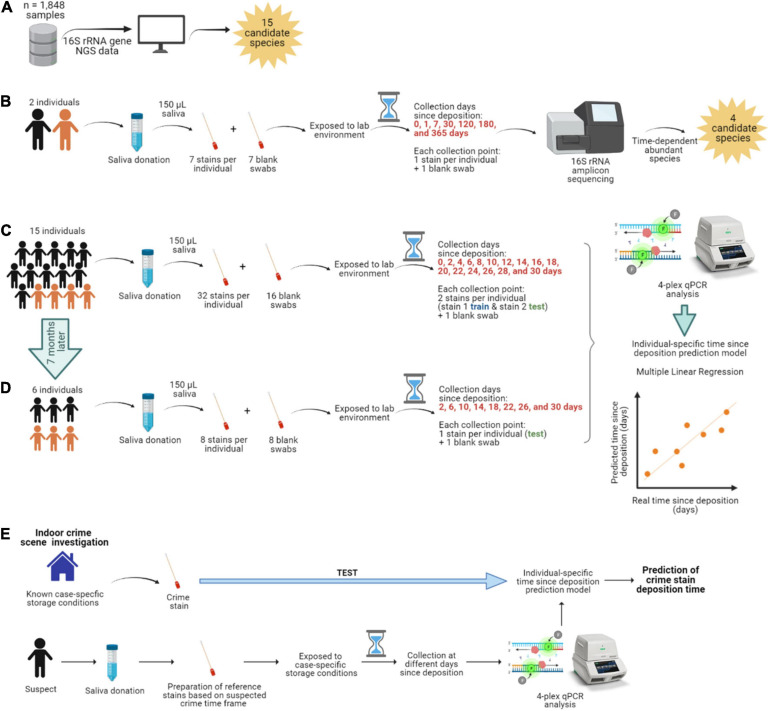
Overview of the study. **(A)** Identification of the most abundant and frequent bacterial species in saliva from publicly available 16S rRNA gene next-generation sequencing (NGS) data. **(B)** Long-term (up to 1 year) time-dependent differentially abundance analysis in saliva stains from two individuals. The individuals are color-coded; black is female, and orange is male. **(C)** Short-term (up to 1 month) targeted analysis of species informative for time since deposition of saliva stains from 15 individuals and **(D)** from six individuals re-sampled 7 months later. **(E)** Hypothesized individualized solution to saliva trace timing as viewed in our data set.

We analyzed the above-mentioned human saliva 16S rRNA gene NGS data to identify the most abundant and frequent bacterial species across individuals included in the two selected studies (Figure 1A). Primer sequences were obtained from the original studies and were removed from the raw sequencing reads using cutadapt (v2.6; Martin, 2011) by setting the minimum-length to 100, to discard processed reads shorter than 100 bp. The resulting FASTQ files were quality-filtered and de-noised using DADA2 (v1.12.1; Callahan et al., 2016). Parameter maxN was set to 0 in the ACS CPS-II/PLCO study to prevent unambiguous nucleotides in the sequencing reads, whereas maxN was set to 1 in the AGP study to avoid too few reads passing the filtering. Parameter maxEE for the maximum number of “expected errors” in the reads was set to 2 in the two studies. Parameter truncLen was set based on the read quality profiles, ensuring to maintain an overlap between forward and reverse reads to be merged later. Following sample inference of true sequence variants, an amplicon sequence variant (ASV) table was constructed and chimeric sequences were removed. At this point, only high-coverage samples (>1,000 reads) were chosen for downstream analysis, resulting in 525 (ACS CPS-II), 452 (PLCO), and 871 (AGP) samples.

To assign taxonomy the ASV table was processed via the *assignTaxonomy* function in DADA2 at taxonomic ranks of interest (phylum, class, order, family, genus, and species). The expanded Human Oral Microbiome Database (eHOMD; v15.2; Escapa et al., 2018) was transformed to a DADA2-compatible training format and used as the reference database. Since the eHOMD database is bacteria-exclusive, the ASV table was further compared against the SILVA SSU r132 database (Quast et al., 2013) to check for sequences assigned to Eukarya, mitochondria, or chloroplasts, which were subsequently filtered out. We additionally filtered out taxa present in less than 0.005% of relative abundance.

Finally, in each study, we ordered the bacterial species according to their relative abundance (number of bacterial species sequencing reads divided by the total number of sequencing reads) and frequency (number of samples in which the bacterial species is reported divided by the total number of samples). As abundant and frequent bacterial species in saliva, we selected the top 15 most common ones across the studies. The processed NGS data in the form of the relative abundance tables of the identified taxa can be found in Supplementary Data Sheet 1.

### Saliva Collection

Sample collection, handling, and subsequent analysis adhered to the Medische Ethische Toestsings Commissie Erasmus MC (MEC-2018–1731). All individuals who donated saliva provided signed informed consent for this study. One individual included in the long-term experiment (individual No 1) was also included in the short-term one (individual No 2), with a time span of 2 years between saliva collection for each of the experiments. In summary, we included 11 females and 5 males, with an average age of 26.3 years and from various ethnic backgrounds, but mainly white Europeans (13/16). Information on our donors’ sex, age, ethnicity, and sample donation can be found in Supplementary Table 1.

In brief, as part of donating saliva, individuals were asked to avoid all of the following for at least 1 h before saliva donation: brushing their teeth, use of mouthwash, eating food, chewing gum, and were asked to only drink non-sparkling water. Individuals were independently asked to collect saliva in their mouth for a minute and spit it into a sterile tube, repeating the process several times until reaching ∼5 mL of saliva. Subsequently, for each individual, we prepared all stains per time point, each consisting of 150 μL of saliva deposited on a sterile swab (PurFlock Ultra 6” sterile standard flock swabs, Puritan, Guilford, ME, United States). Therefore, for each individual, all saliva stains were individual samples, even though they were collected at the same time point. As a substrate, we chose sterile swabs that are routinely used in forensics, for example, to collect suspected saliva stains from objects found at the crime scene for molecular analysis. The sterile nature of the swabs also allowed us to conclude that there was an absence of microbial contamination before the saliva was deposited.

### Dried Saliva Stains

With the exception of the fresh (t0) saliva swabs that were processed straightaway, the prepared swabs were dried and directly exposed to our laboratory’s environment apart from each other, for a specific time prior to bacterial DNA isolation. The swabs were stored at standard room temperature (20–25°C), with relative humidity (30-50%), and daily ambient light (8-11 h). Ambient light included both artificial and natural light sources as the swabs were placed 4 m away from a window (though not directly hit by the sun). First, for the long-term time-dependent bacterial composition analysis, saliva was collected from two individuals (Figure 1B). To sufficiently cover selected time points over a 1-year period, seven saliva swabs were prepared per individual (single sample replicates per time point) and processed at day 1 (t1), 7 (t2), 30 (t3), 120 (t4), 180 (t5), and 365 (t6) after deposition. Second, for the short-term time-dependent bacterial marker analysis, saliva was collected from 15 individuals (Figure 1C). To sufficiently cover selected time points over a 1-month period, 32 saliva swabs were prepared per individual (double replicates per time point); with the exception of one individual (No 1) for which there was insufficient volume of saliva to prepare the last time point. In this case, dried saliva swabs were processed at day 2 (t1), 4 (t2), 6 (t3), 8 (t4), 10 (t5), 12 (t6), 14 (t7), 16 (t8), 18 (t9), 20 (t10), 22 (t11), 24 (t12), 26 (t13), 28 (t14), and 30 (t15) after deposition. Additionally, six of these fifteen individuals also donated saliva 7 months after the first collection date (Figure 1D). For this, eight saliva swabs were prepared per individual (single replicates per time point) and processed at day 2 (t1), 6 (t2), 10 (t3), 14 (t4), 18 (t5), 22 (t6), 26 (t7), 28 (t8 for individual No 1), and 30 (t8 for the rest of individuals) after deposition, corresponding to 1-month time frame as in the first collection date. Additionally, swabs with no biological material were prepared as background blanks in both the long-term and short-term experiments and were exposed and processed in parallel at the same time points as the dried saliva stains.

### Bacterial DNA Isolation and Quantification

Bacterial DNA isolation was performed with the QIAamp DNA Mini Kit (Qiagen, Germany) following the buccal swab spin protocol to simplify the isolation of DNA from human saliva samples deposited on a swab. We chose a kit that can co-isolate both the bacterial and human DNA present in the sample to simultaneously allow for STR profiling, necessary to identify the sample donor, and therefore, increasing the forensic applicability of the proposed approach. After appropriate optimization, we slightly modified the manufacturer’s instructions for maximizing DNA yield. More specifically, the incubation time in step four was increased from 10 to 30 min, the elution was performed with nuclease-free water using a reduced 50 μL elution volume, spin columns were incubated for 5 min at room temperature following the addition of nuclease-free water and before centrifugation, the centrifugation time and speed were increased to 2 min and 12,000 rpm, and finally, a second elution step using the eluate was added. Isolated bacterial DNA was quantified with the Femto^TM^ Bacterial DNA Quantification kit (Zymo Research, Irvine, CA, United States) following the manufacturer’s instructions on a CFX38 Touch^TM^ Real-Time PCR System (Bio-Rad, Hercules, CA, United States).

### Library Preparation and Sequencing

For the long-term time-dependent bacterial composition analysis, we sequenced the obtained bacterial DNA from the dried saliva stains of the two individuals (*N* = 14) as well as the background controls (*N* = 7). To also assess the performance of the workflow we sequenced one negative control sample, one smart control (SC) sample for monitoring the library construction process and potential introduced contamination, and one positive control – a commercial microbial community DNA standard sample (ZymoBIOMICS^TM^ Microbial Community DNA Standard, ZymoResearch). Library preparation was performed using the QIAseq 16S/ITS Panel Kit (Qiagen) for sequencing the V4–V5 region of the 16S rRNA bacterial gene. Library quality control was performed with the Agilent 2100 Bioanalyzer (Agilent Technologies, Palo Alto, CA, United States) using a high sensitivity DNA chip following the manufacturer’s instructions. Library quantification was performed using the KAPA Library Quantification Kit (Kapa Biosystems, Inc. Wilmington, MA, United States) following the manufacturer’s instructions on a CFX384 Touch^TM^ Real-Time PCR System (Bio-Rad). Libraries were diluted down to 2 nM, or the highest possible concentration in case the library concentration was <2 nM, and pooled together for 2 × 276 bp paired-end sequencing on a MiSeq platform using the MiSeq v3 Reagent Kit (Illumina, San Diego, CA, United States).

### Long-Term Time-Dependent Differential Bacterial Abundance Analysis

We performed differential abundance analysis to identify changes in the relative abundance of bacterial species over time in the 16S rRNA gene NGS data derived from the long-term dried saliva stains. Phased primer sequences were removed from the raw sequencing reads using a custom Python script. Subsequent filtering, de-noising, ASV table construction, and taxonomy annotation were carried out as previously described for the publicly available saliva microbiome data sets (section “Most abundant and frequent bacterial species in saliva”). We chose gneiss (Morton et al., 2017) for the differential abundance analysis since it acknowledges the compositional nature of microbiome data. Based on this compositional nature it is only possible to infer relative, but not absolute, abundance changes with time, since the abundance change of one species influences the abundance changes in the other species. Gneiss was run using the q2-gneiss plugin in QIIME2 (v.2019.10; Bolyen et al., 2019). Input data comprised of a microbial profile sub-selection of the 15 most abundant and most frequent bacterial species in saliva as previously identified (section “Most abundant and frequent bacterial species in saliva”). First, a bifurcating tree was built relating bacterial species to each other based on how they co-occur by using Ward’s hierarchical clustering via the *correlation-clustering* command. Each balance (internal nodes in the tree) is calculated by taking the log ratio of geometric means of subtrees via the *ilr-transform* command. Each balance is indicated as “*y*” followed by an ordinal number, being *y0* the first balance in the root of the constructed tree. The taxa on one side of the balance are termed as numerators and on the other side as denominators. Each log ratio’s numerical value depends on the balance between the numerator’s and denominator’s taxa and can be either positive, negative, or null. Differences in the log ratio balances can be compared between sample groups to infer relative changes in the microbial composition. These log-transformed balances were used to construct a multivariate response linear model using the time since deposition and the individual ID as covariates using the *ols-regression* command, where 10-fold cross validation of 10 partitions showed no overfitting. The regression summary showed the contributions of the covariates to the abundances of the selected bacterial species. Balances significantly affected by the covariates were determined with a *p* value cutoff at 0.05 after Bonferroni correction. These *p* values were based on relative, rather than absolute, values resulting from inter-dependent taxa. Significant balances for time since deposition but not for individual ID were selected as the most informative for this study and used to analyze the informative bacterial species via qPCR in the short-term dried saliva stains.

### 4-Plex qPCR Assay Design and Optimization

Based on the differential abundance analysis results, four bacterial species were selected for qPCR analysis of the short-term dried saliva stains. The selected species were *Fusobacterium periodonticum, Haemophilus parainfluenzae, Veillonella dispar, and Veillonella parvula.* We aimed to design a suitable 4-plex qPCR assay based on TaqMan probe technology that would allow for the simultaneous analysis of all four selected bacterial species using species-specific primers that target single-copy genes. For *F. periodonticum*, *V. dispar*, and *V. parvula* we chose the beta subunit of RNA polymerase gene (rpoB) as the target gene; for *H. parainfluenzae* we chose the translation initiation factor IF-2 gene (infB).

A literature search was conducted to find previously designed suitable primers, resulting in the reverse primers for *V. dispar* and *V. parvula* (Mashima et al., 2016). The rest of the primer sequences as well as the probe sequences were manually designed using the PrimerQuest Tool (Integrated DNA Technologies, IDT, Coralville, IA, United States). The fluorescent dyes labeled to the 5′-end of the probe sequences were: 6-carboxyfluorescein (6-FAM) for *F. periodonticum*, cyanine 5 (Cy5) for *H. parainfluenzae*, Texas red-615 (TEX-615) for *V. dispar*, and hexachloro-fluorescein (HEX) for *V. parvula.* To test primer pair specificity, each pair was compared against the nucleotide collection database from the NCBI using Primer BLAST. The Autodimer software (Vallone and Butler, 2004) was also used to assess the potential formation of primer dimers and hairpins under our experimental conditions. Final primer and probe sequences are summarized in Supplementary Table 2.

The 4-plex qPCR assay was developed based on the CFX384 Touch^TM^ Real-Time PCR System (Bio-Rad). The assay was optimized according to various parameters including annealing temperature and primer/probe concentrations. The optimal oligo concentrations varied for each bacterial target and were determined as follows (primers/probe): *F. periodonticum* (0.7/0.5 μM), *H. parainfluenzae* (0.6/0.5 μM), *V. dispar* (0.2/0.05 μM), *and V. parvula* (0.9/0.5 μM). Synthetic double stranded DNA fragments (gBlocks, IDT) for each of the bacterial target gene fragments were used as standard samples (positive controls; Supplementary Table 2). Concentrations were converted to copy numbers by using the formula:

(C)*(M)*(1*10-15⁢mol/fmol)*(A⁢v⁢o⁢g⁢a⁢d⁢r⁢o⁢s′⁢n⁢u⁢m⁢b⁢e⁢r)=c⁢o⁢p⁢y⁢n⁢u⁢m⁢b⁢e⁢r/μ⁢L.

where *C* is the concentration of the gBlock gene fragment in ng/μL and *M* is the molecular weight in fmol/ng. gBlocks were mixed in known concentrations ranging from 125,000 down to 61 copies per bacterial target gene fragment. The assay was performed in a 20 μL reaction in triplicate, including 10 μL of iQ Multiplex Powermix (Bio-Rad), 4 μL of each primer (forward and reverse), and probe mix (5X), 1 μL of 25 μM of MgCl_2_ (Thermo Fisher Scientific, Waltham, MA, United States), 0.5 μL of 20 mg/mL of bovine serum albumin (New England Biolabs, Ipswich, MA, United States), 1 μL of bacterial DNA (corresponding to 2 ng) and 3.5 μL of nuclease-free water. The thermocycling program included an initial denaturation and polymerase activation step at 95°C for 3 min, followed by 35 PCR cycles of 95°C for 10 s and an extension step of 60°C for 45 s.

### qPCR Data Analysis

Using our developed and optimized 4-plex qPCR assay we analyzed the short-term dried saliva stains of 15 individuals. The standard samples with known concentrations per bacterial target gene fragment (gBlocks) were used to create the best-fitted linearity curve. The efficiency of each qPCR assay was calculated from the slope of the serially diluted standard samples according to the equation (Kubista et al., 2006):

$$E=10^{-\left(1/\mathrm{slope}\right)}.$$

For each reaction, we obtained the quantification cycle (*Cq*) value, the point at which fluorescence above the threshold level is detectable. To standardize, the threshold was set to 100 relative fluorescence units (RFU) for all reactions and fragments. The copy number (cn) for each bacterial target gene fragment was calculated according to the equation:

$$c\,n=e^{-C\,q}.$$

Since we target single-copy genes, reported copy numbers can be translated to bacterial cell counts. Between-plate variation was removed using the Factor-qPCR tool (Ruijter et al., 2015) in the stains produced at the first donation time point and 7 months later for six individuals. We set the qPCR plate ID as the variable causing the variation to be removed, while the bacterial marker and time since deposition as the variables for which preserve their effects. In some cases, the between-plate correction resulted in negative count values.

### Short-Term Time-Dependent Bacterial Analysis

We aimed to investigate the statistical relationship between the time since deposition and the four selected bacteria cell counts in the short-term dried saliva stains. Since the focus was on dried saliva stains, fresh (t0) samples were excluded from this analysis. Sample duplicates collected at each time point for each donor were analyzed independently from each other to assess the magnitude of sample variation. For each analyzed stain qPCR triplicates were considered as separate samples to account for potential reaction variation. Various linear regression models were built using the *lm()* function in the lme4package (v.1.1.20; Bates et al., 2015) in R (v.3.6.1 [2019-07-05]). The linear models were based on the functions below, where C refers to the bacterial cell count, I to the individual, S to the bacterial species, and finally, *T* to the time since deposition (in days). Interactions between variables are indicated with an asterisk (^∗^). The statistical relationship between each bacterial species cell count for each individual and the time since deposition was calculated based on the function:

lm(C∼T).

The statistical relationship between the four species cell count and the time since deposition, species, and their interaction for each individual was calculated based on the function:

lm(C∼T*S).

The statistical relationship between each species cell count and the time since deposition, individual, and their interaction was calculated based on the function:

lm(C∼T*I).

Finally, the statistical relationship between the four species cell count and the time since deposition, species, individual, and their interaction was calculated based on the function:

lm(C∼T*S+T*I).

Adjusted *R*^2^ and *p* values were evaluated for all the linear models. Sample variation was assessed by testing for equality between the coefficients in the linear regression models of each of the sample duplicate sets using the Chow test implemented in the gap R-package (v.1.2.2; Zhao, 2007). Significant *p* values were determined with a value cutoff at 0.05 following Benjamini-Hochberg correction. All plots were generated with the ggplot2 R-package (v.3.3.2; Hadley, 2009).

### Time Since Deposition Prediction Modeling

We further investigated the possibility to predict the time since deposition in the short-term dried saliva stains. We once again excluded fresh (t0) samples based on the notion that it is not feasible to collect a purely fresh saliva sample at a real crime scene. We first attempted a generalized time since deposition prediction model based on random forest (RF) regression using the randomForest R-package (v.4.6.14; Liaw and Wiener, 2002). To evaluate the generalizability of this approach and to avoid prediction biases, we built a model based on the average detected microbial DNA cell counts of the four targeted species per time point. We then used data from all time points from 14 of the analyzed individuals as the training set, while keeping all the time points of the remaining individual as the testing set. By this, the tested individual was not present in the training set to mimic real-life applications. We repeated this process 15 times given the 15 individuals in our data set. The 15 RF models were based on 5-fold cross-validation, which was repeated three times, and 500 trees with the four variables (targeted bacterial species) were sampled at each split. NA values were replaced with column medians using the *na.roughfix* command. The average performance of the generalized RF models was assessed using the mean absolute error (MAE), which measures the discrepancies between predicted and real values according to the formula below:

$$M\,A\,E=\frac{1}{n}\,\sum_{i=1}^{n}\left|y\,i-y\right|$$

where *n* is the total number of data points, *y*_*i*_ is the real value and *y* is the predicted value. Pearson’s correlation was used to calculate the correlation (*r*) between real and predicted values. MAE and *r* were calculated with the Metrics R-package (v.0.1.4; Hamner et al., 2012).

In the individualized modeling approach, the sample duplicates collected at each time point for each donor were considered separately; namely, one duplicate was used as the training sample, while the other was used as the testing sample, mimicking a potential future forensic scenario of having both reference and crime scene samples. As predictors, we chose the bacterial cell counts of the four selected species at each time point. Multiple linear regression models were built using the *lm()* function in the lme4 R-package (Bates et al., 2015) based on the function below:

$$lm\left(T∼C_{1}+C_{2}+C_{3}+C_{4}\right)$$

where *C* refers to each bacterial species cell counts and *T* to the time since deposition in days. Additionally, the follow-up dried saliva stains of the selected six individuals were also analyzed as testing samples. In this case, as time since deposition predictors, we did not only consider the bacterial cell counts for the four selected species, but all the possible combinations of also one, two, and three predictor species to select the model with the lowest error for each individual. The donor-specific prediction models were evaluated based on the adjusted *R*^2^ and *p* values, where significant *p* values were determined with a value cutoff at 0.05 following Benjamini-Hochberg correction. The average model performances were assessed using the MAE and Pearson’s correlation was used to calculate the correlation (*r*) between real and predicted values. All plots were generated with the ggplot2 R-package (v.3.3.2; Hadley, 2009). The processed qPCR data used to build and test the prediction models can be found in Supplementary Data Sheet 1.

## Results

### Selection of Most Abundant and Most Frequent Bacterial Species in Human Saliva From Large 16S rRNA Gene Sequencing Data

Publicly available 16S rRNA gene NGS data from 1,848 human saliva samples were analyzed to identify the most abundant and most frequent bacterial species across the studies they were retrieved from (Wu et al., 2016; McDonald et al., 2018). A total of 10,326,403 sequencing reads were retrieved from the ACS CPS-II/PLCO study and 31,046,365 sequencing reads from the AGP study. In the ACS CPS-II/PLCO study, 218 bacterial species from 35 families were identified, while in the AGP study 471 bacterial species from 88 families were found.

**FIGURE 2:**
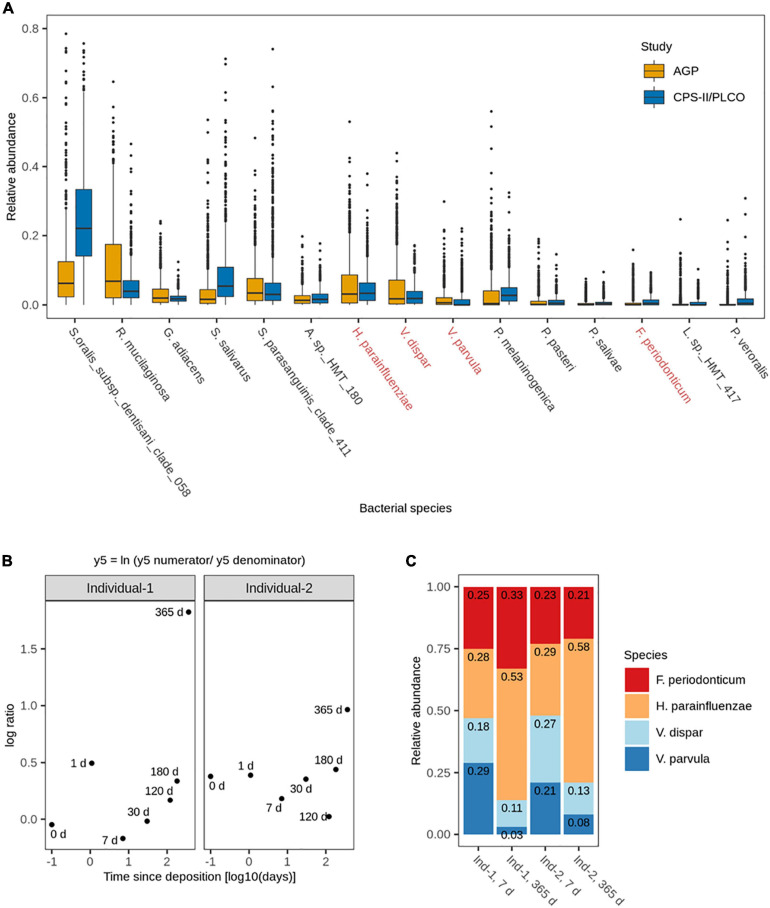
**(A)** Relative abundances of the 15 most abundant and frequent bacterial species in human saliva from adults across the analyzed publicly available 16S rRNA gene NGS data sets (*N* = 1,848). Highlighted in red are the four bacterial species subsequently included in the 4-plex qPCR assay. **(B)** Log ratio balance y5, significantly different for the time since deposition in the *de-novo* generated 16S rRNA gene NGS data from the long-term (up to 1 year) dried saliva stains. Time since deposition days (*x*-axis) were log-transformed to facilitate the visualization of the earliest time points. Each dot represents a long-term dried saliva stain with its corresponding time since deposition. **(C)** Relative abundances of the four bacterial species from balance y5 (*Fusobacterium periodonticum, Haemophilus parainfluenzae, Veillonella dispar*, and *Veillonella parvula)* in the long-term (up to 1 year) dried saliva stains at day 7 and day 365 since stain deposition for both analyzed individuals.

We then selected the top 15 most abundant and most frequent bacterial species from a total of 10 families that were common across these studies, namely *Actinomyces sp. HMT 180*, *Fusobacterium periodonticum*, *Granulicatella adiacens, Haemophilus parainfluenzae, Leptotrichia sp. HMT 417, Porphyromonas pasteri, Prevotella melaninogenica, Prevotella salivae, Prevotella veroralis, Rothia mucilaginosa, Streptococcus oralis subs. dentisani clade 058, Streptococcus parasanguinis clade 411, Streptococcus salivarus*, *Veillonella dispar*, and *Veillonella parvula* (Figure 2A). These 15 identified common species accounted for 66.0% (6,817,142) of the sequencing reads in the ACS CPS-II/PLCO study and 55.1% (17,095,402) of the sequencing reads in the AGP study. In the ACS CPS-II/PLCO study, *S. oralis subs. dentisani clade 058* was the most abundant species accounting for 24.9% (2,567,719) of the reads, whereas *L. sp. HMT 417* was the least abundant accounting for 0.59% (61,475) of the reads. In the AGP study, *R. mucilaginosa* was the most abundant species accounting for 10.7% (3,305,923) of the reads, whereas *P. salivae* was the least abundant accounting for 0.47% (146,083) of the reads. Overall, these 15 common species were similar in abundance across the analyzed studies, with the exception of *S. oralis subs. dentisani clade 058*, which was markedly more abundant in the ACS CPS-II/PLCO than in the AGP study (24.9% *vs.* 9.1% of total reads), and *R. mucilaginosa* which was more abundant in the AGP than in the ACS CPS-II/PLCO study (10.6% *vs.* 5.4% of total reads). The most frequent species was *S. oralis subs. dentisani clade 058*, which was present in 97.0% (948) of the ACS CPS-II/PLCO study’s individuals, and found in 95.2% (829) of the AGP study’s individuals. The less frequent species in the ACS CPS-II/PLCO study was *L. sp. HMT 417* present in 46.2% (451) of the individuals, whereas in the AGP study it was *P. veroralis* present in 33.9% (295) of the individuals.

### Selection of Bacterial Species With Time-Dependent Relative Abundance in Long-Term Dried Saliva Stains Using *de-novo* 16S rRNA Gene Sequencing

We then analyzed the dried saliva stains produced from two individuals and exposed to indoor conditions for different time periods of up to 1 year. The obtained 16S rRNA microbial profiles were distinct from the background blanks (empty swabs) for each time point, indicating a low level of bacterial contamination (Supplementary Figure 1). We then extracted the data of the 15 most abundant and most frequent bacterial species identified in our previous *in silico* analysis (Figure 2A) to identify for which species their relative abundance significantly changed over time. The fit of the overall multivariate response linear model was *R*^2^ = 0.21, with the time since deposition accounting for 7% and the person accounting for 15% of the bacterial variation.

A total of 13 log ratio balances (from y0 to y12) were generated as internal nodes in the built tree. Log ratio balances y4 (*p* = 0.008), y5 (*p* = 0.004), and y7 (*p* = 0.022) were significantly different for the time since deposition (Table 1). Balance y4 was composed of *A. sp. HMT 180, S. oralis subsp. dentisani clade 058, S. parasanguinis clade 411, P. pasteri, P. melaninogenica*, and *P. veroralis* as numerator’s taxa; and *F. periodonticum, P. melaninogenica, V. dispar*, and *V. parvula* as denominator’s taxa. Balance y5 was composed of *F. periodonticum, H. parainfluenzae*, and *V. dispar* as numerator’s taxa; and *V. parvula* as denominator’s taxa. Balance y7 was composed of *F. periodonticum*, and *H. parainfluenzae* as numerator’s taxa; and *V. dispar* as denominator’s taxa. It has to be noted that balance y7 is a subdivision of balance y5 numerator (Table 1). An overview of the generated log ratio balances, intercept, and *p* values for the time since deposition and individual person can be found in Supplementary Table 3.

**TABLE 1:** Significant log ratio balances for time since deposition in the differential abundance analysis.

Balance	Bacterial species	*p* value; time since deposition	*p* value; individual person
y4_numerator_	*A. sp. HMT 180* *S. oralis subsp. dentisani clade 058* *S. parasangunis clade 411* *P. pasteri* *P. melaningenica* *P. veroralis*	0.008	0.390
y4_denominator_	*F. periodonticum* *P. melaningenica* *V. dispar* *V. parvula*		
y5_numerator_	*F. periodonticum* *H. parainfluenzae* *V. dispar*	0.004	0.911
y5_denominator_	*V. parvula*		
y7_numerator_	*F. periodonticum* *H. parainfluenzae*	0.022	0.022
y7_denomi__nator_	*V. dispar*		

*The top 15 most abundant and frequent bacterial species in human saliva were sub-selected from the *de-novo* generated 16S rRNA gene NGS data obtained from the long-term dried saliva stains. Each balance is composed of the numerator’s bacterial taxa and the denominator’s bacterial taxa.*

For this study, we sub-selected the log ratio balance y5 as our reference because of its strongest significant time dependency (*p* = 0.004) in both individuals. We preferred y5 over its subdivision y7 since balances toward the root of the tree capture more information as they contain more tree tips. Furthermore, three of the four bacterial species in y5 were also present in y4, which also showed a strong significant time dependency (*p* = 0.008), albeit less strong than y5 (Table 1). For both individuals, there was a similar pattern in the log ratio evolution of balance y5 through time since saliva stain deposition, though the rate of change was individual-specific (Figure 2B). From 7 to 365 days, the general trend was the increase of the log ratios’ values for both individuals. Looking at the relative abundances of the four species from balance y5 at day 7 and day 365 since deposition we observed that for *H. parainfluenzae* relative abundance increased in both individuals; for *V. dispar* and *V. parvula* relative abundances decreased in both individuals; and for *F. periodonticum* relative abundance increased in individual 1, whereas it slightly decreased in individual 2 (Figure 2C). Based on these results, the four species composing balance y5 were selected for developing a 4-plex qPCR assay for their targeted analysis in the short-term dried saliva stains. Parallel to the NGS analysis, the relative abundance of the four selected species in the background (blank) swabs was very low (≤1%); on average (mean ± standard deviation), as follows: *F. periodonticum* (0.008 ± 0.010), *H. parainfluenzae* (0.010 ± 0.009), *V. dispar* (0.005 ± 0.006), and *V. parvula* (0.006 ± 0.009).

### Relationship Between Bacterial Abundance and Time Since Deposition in Short-Term Dried Saliva Stains Using Multiplex qPCR

Dried saliva stains from 15 individuals up to 1 month since deposition were analyzed using the 4-plex qPCR assay we developed. Parallel to the qPCR analysis, no signal above the set threshold was reported in the background (blank) swabs forany of the four bacterial markers and time points. The qPCR results obtained from the fresh (t0) samples confirmed that the four selected bacterial species were abundant and frequent in the saliva of all 15 individuals, although we observed high inter-individual variation within and between species. For each of the four species, the average and standard deviation (mean ± SD), as well as the minimum and maximum value (range), of qPCR-derived cell counts in 1 μL of isolated bacterial DNA solution (equivalent to 2 ng of total bacterial DNA) were as follows: *F. periodonticum* (23,386 ± 24,598; range 2,698–105,567), *H. parainfluenzae* (83,854 ± 80,412; range 13,167–331,667), *V. dispar* (20,071 ± 27,406; range 399–91,937), and *V. parvula* (9,825 ± 21,354; range 309–92,347). A figure of the bacterial cell count distribution in fresh saliva samples for each of the four species can be found in Supplementary Figure 2.

**FIGURE 3:**
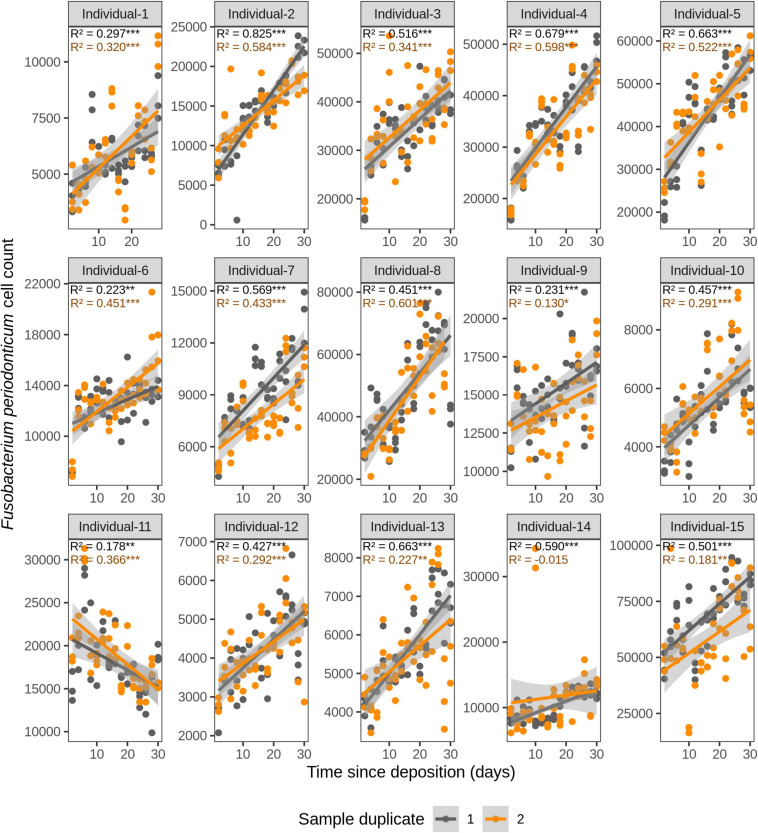
Time-dependency of qPCR-based *Fusobacterium periodonticum* cell count for each individual in the short-term (up to 1 month) dried saliva stains from 15 individuals. Sample duplicate set 1 is indicated in gray and sample duplicate set 2 in orange. Each dot represents a qPCR-run sample triplicate. *R*^2^ values indicate the variation explained by the time since deposition in the bacterial cell count. Asterisks indicate the significance level of the Benjamini-Hochberg corrected *p* values as follows: 0.001 “***”, 0.01 “**”, and 0.05 “*”.

**FIGURE 4:**
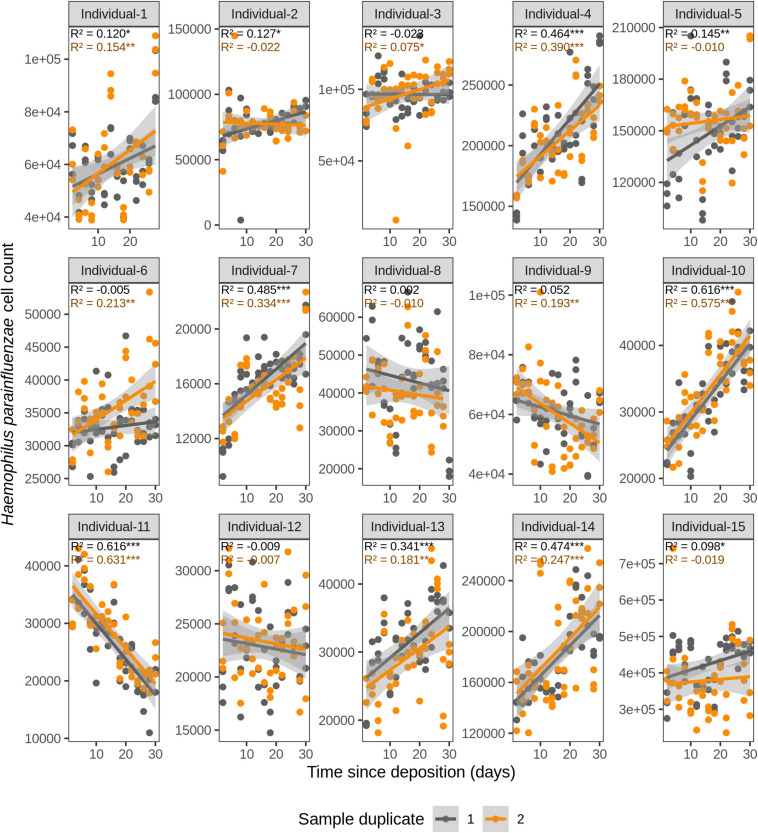
Time-dependency of qPCR-based *Haemophilus parainfluenzae* cell count for each individual in the short-term (up to 1 month) dried saliva stains from 15 individuals. Sample duplicate set 1 is indicated in gray and sample duplicate set 2 in orange. Each dot represents a qPCR-run sample triplicate. *R*^2^ values indicate the variation explained by the time since deposition in the bacterial cell count. Asterisks indicate the significance level of the Benjamini-Hochberg corrected *p* values as follows: 0.001 “***”, 0.01 “**”, and 0.05 “*”.

**FIGURE 5:**
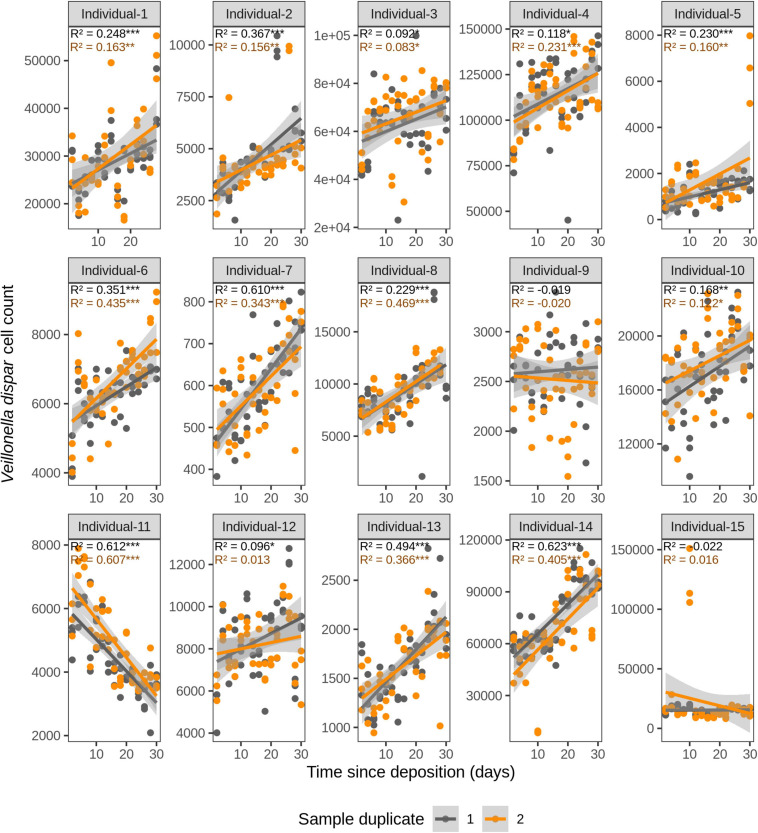
Time-dependency of qPCR-derived *Veillonella dispar* cell count for each individual in the short-term (up to 1 month) dried saliva stains from 15 individuals. Sample duplicate set 1 is indicated in gray and sample duplicate set 2 in orange. Each dot represents a qPCR-run sample triplicate. *R*^2^ values indicate the variation explained by the time since deposition in the bacterial cell count. Asterisks indicate the significance level of the Benjamini-Hochberg corrected *p* values as follows: 0.001 “***”, 0.01 “**”, and 0.05 “*”.

**FIGURE 6:**
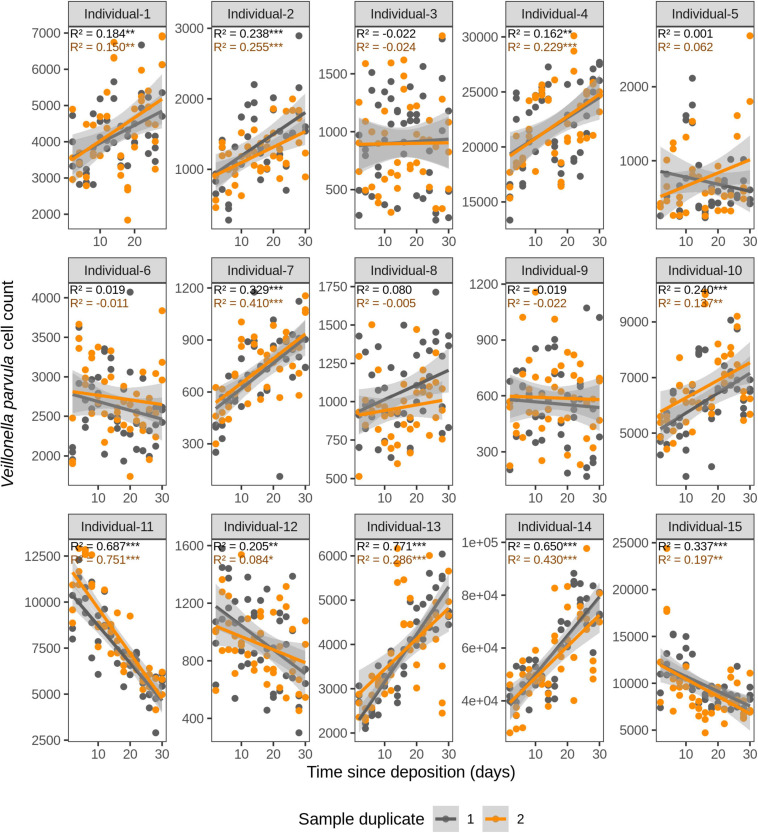
Time-dependency of qPCR-derived *Veillonella parvula* cell count for each individual in the short-term (up to 1 month) dried saliva stains from 15 individuals. Sample duplicate set 1 is indicated in gray and sample duplicate set 2 in orange. Each dot represents a qPCR-run sample triplicate. *R*^2^ values indicate the variation explained by the time since deposition in the bacterial cell count. Asterisks indicate the significance level of the Benjamini-Hochberg corrected *p* values as follows: 0.001 “***”, 0.01 “**”, and 0.05 “*”.

We first investigated the time dependency of each of the four species in each individual in the dried saliva stains ranging from 2 days (t1) up to 30 days (t15) since deposition. The Chow test for equality showed no significant differences in the great majority of the compared time point swab duplicates’ regressions. Exceptions were the univariate linear regressions for *F. periodonticum* in individual 2 (*p* = 0.010), individual 7 (*p* = 0.005), and individual 15 (*p* = 0.020) and for *H. parainfluenzae* in individual 6 (*p* = 0.020). We observed high inter-individual differences in terms of the amount of variation explained by time for each species’ cell count (Figures 3–6). For example, in individual 5 the variation explained for *F. periodonticum* cell count was high in both duplicates (*R*^2^ = 0.663, *p* = 1.06E–10 in duplicate 1; and *R*^2^ = 0.522, *p* = 6.92E–08 in duplicate 2; Figure 3). However, the variation explained for *V. parvula* was much lower, even close to zero (*R*^2^ = 0.001, *p* = 0.374 in duplicate 1; and *R*^2^ = 0.062, *p* = 0.092 in duplicate 2; Figure 6). The univariate regression results including *R*^2^ values, BH-corrected *p* values, and significance testing can be found in Supplementary Table 4.

For *F. periodonticum*, the qPCR-derived cell count increased over time for most individuals, except for individual 11, although at different rates in the different individuals. The average and standard deviation fold-change between day 2 (t1) and day 30 (t15) were 2.0 ± 0.6, range 1.1–3.3. The highest time-dependent bacterial increase was reported for individual 2 (*R*^2^ = 0.825, *p* = 2.59E–14, sample duplicate 1; Figure 3). For *H. parainfluenzae*, the time-dependent behavior varied in an individual-specific manner meaning either increasing (individuals 1, 4, 5, 6, 7, 10, 13, and 14), decreasing (individuals 9 and 11), or barely changing (individuals 2, 3, 8, 12, and 15; Figure 4). For *V. dispar*, the cell count increased with time in the majority of the individuals at different rates, except individual 11. The average and standard deviation fold-change between day 2 (t1) and day 30 (t15) were 1.7 ± 1.1, range 1.1–7.0. The highest time-dependent bacterial increase was reported for individual 14 (*R*^2^ = 0.623, *p* = 8.70E–10, sample duplicate 1; Figure 5). Lastly, for *V. parvula*, cell count increased with time for some individuals (individuals 1, 2, 4, 7, 10, 13, and 14), whereas for others it decreased (individuals 11, 12, and 15) or barely changed (individuals 3, 5, 6, and 9; Figure 6).

We next investigated the time dependency of the four bacterial species altogether in each individual (Table 2). The variation explained by time in the four species cell count varied among individuals, but it was very high in the majority of them with *R*^2^ values ranging between 0.865 and 0.979 (*p* = <2.20E–16). The individual with the highest variation explained on average was individual 5 (*R*^2^ = 0.964 in sample duplicate 1, *R*^2^ = 0.979 in sample duplicate 2, *p* = <2.20E–16). The individual with the lowest variation explained on average was individual 8 (*R*^2^ = 0.874 in duplicate 1, *R*^2^ = 0.908 in duplicate 2, *p* = <2.20E–16). We also investigated the time dependency of each bacterial species considering the 15 individuals altogether (Table 2). The variation explained by time for each of the four bacterial species cell count in all individuals together was very high and significant. The strongest time dependency variation on average (*R*^2^ in duplicate 1, *R*^2^ in duplicate 2, and *p* value) was observed for *V. parvula* (*R*^2^ = 0.969, *R*^2^ = 0.948, *p* = <2.20E–16), followed by *H. parainfluenzae* (*R*^2^ = 0.964, *R*^2^ = 0.920, *p* = <2.20E–16), *V. dispar* (*R*^2^ = 0.959, *R*^2^ = 0.906, *p* = <2.20E–16), and finally *F. periodonticum* (*R*^2^ = 0.941, *R*^2^ = 0.889, *p* = <2.20E–16). Finally, we investigated the time dependency of the four bacterial species together in the 15 individuals altogether (Table 2) resulting in a significant variation, explained in the data set of *R*^2^ = 0.544 in duplicate 1 and *R*^2^ = 0.548 in duplicate 2, *p* = <2.20E–16.

**TABLE 2:** Time-dependency of the bacterial marker cell counts in the dried saliva stains exposed to indoor conditions up to 1 month for sample duplicate sets 1 and 2.

		Sample duplicate 1		Sample duplicate 2	
			
Linear regression analysis		*R*^2^	*p* value	*R*^2^	*p* value
lm(C ∼ T*S)	Individual 1	0.922	<2.2E–16***	0.865	<2.2E–16***
	Individual 2	0.952		0.948	
	Individual 3	0.940		0.907	
	Individual 4	0.955		0.966	
	Individual 5	0.964		0.979	
	Individual 6	0.968		0.962	
	Individual 7	0.973		0.966	
	Individual 8	0.874		0.908	
	Individual 9	0.965		0.946	
	Individual 10	0.953		0.950	
	Individual 11	0.912		0.927	
	Individual 12	0.936		0.946	
	Individual 13	0.968		0.937	
	Individual 14	0.959		0.905	
	Individual 15	0.968		0.902	
lm(C ∼ T*I)	*F. periodonticum*	0.941		0.889	
	*H. parainfluenzae*	0.964		0.920	
	*V. dispar*	0.959		0.906	
	*V. parvula*	0.969		0.948	
lm(C ∼ T*I + T*S)	Overall	0.546		0.551	

lm(C ∼ T*S): time-dependency of the four selected bacterial species altogether in each individual, lm(C ∼ T*I): time-dependency of each selected bacterial species considering the 15 individuals altogether, and lm(C ∼ T*I + T*S): time-dependency of the four selected bacterial species and 15 individuals altogether. *R*^2^ values indicate the variation explained by the time since stain deposition in the qPCR-derived bacterial cell counts. Asterisks indicate the significance level of the Benjamini-Hochberg corrected *p* values as follows: 0.001 “***”, 0.01 “**”, and 0.05 “*”.

### Estimating the Time Since Deposition of Dried Human Saliva Stains Based on Bacterial DNA

**FIGURE 7:**
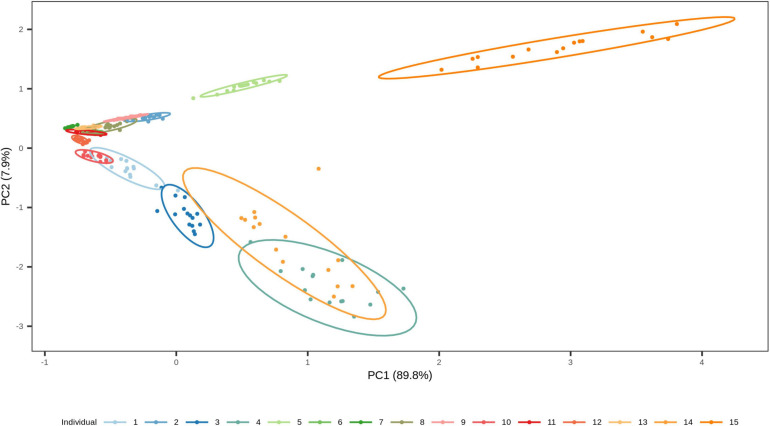
Two-dimensional plot from principal component analysis (PCA) of the short-term dried saliva stains from 15 individuals. Projection of the counts of our four targeted species in the first two PCs together explains 97.7 % of the total variation. The stains cluster based on the individual, which highlights the high inter-individual variation in the data set.

We finally investigated the possibility of estimating the time since deposition of the dried saliva stains exposed to indoor short-term conditions of up to 1 month, using a generalized RF regression model. The correlation between real and predicted time since deposition values were very low (*r* = 0.11; Supplementary Figure 3) and the average MAE was 8 days. The real and predicted times since deposition for each individual are summarized in Supplementary Table 5. From these predicted values we deduced that the generalized approach was unable to discriminate between early and late times since deposition in the analyzed interval of 1 month. For instance, in individual 4 for whom all times since deposition were predicted as either 20 or 21 days and individual 7 for whom all times since deposition were predicted as 16, 17, or 18 days (Supplementary Table 5). Hence, the time-dependent variation in the four targeted bacterial species were surpassed by the high inter-individual variation in our data set. The high inter-individual variation can be observed using principal component analysis (PCA; Figure 7) where the saliva stains cluster based on the individual. Hence, because of the high inter-individual variation we observed, as described in section “Relationship between bacterial abundance and time since deposition in short-term dried saliva stains using multiplex qPCR,” which limited the implementation of a generalized model in our data set, we built individual-specific models.

This individual-specific modeling approach enabled us to present an individualized solution to saliva trace timing in our data set, where the model training and testing data are obtained from the individual’s reference saliva stored under the same environmental conditions for a specific time period (Figure 1E). We hypothesize that for certain indoor crimes where environmental conditions are rather stable, various parameters (temperature, humidity, etc.) could be measured at the crime scene when the stain is collected and applied to the reference saliva stains used to generate the model underlying data with flexibility for the time window. Consequently, in our experiments, we applied the same environmental parameters to saliva samples stored up to 1 month that we used for model training and model testing. The *R*^2^ and BH-corrected *p* values of each individual-specific model can be found in Supplementary Table 6. Overall, the average model fit was *R*^2^ = 0.752. The best model fit was obtained for individual 11 (*R*^2^ = 0.921, *p* = 4.80E–05), whereas for individual 1 the model barely fit (*R*^2^ = 0.178, *p* = 0.233).

**FIGURE 8:**
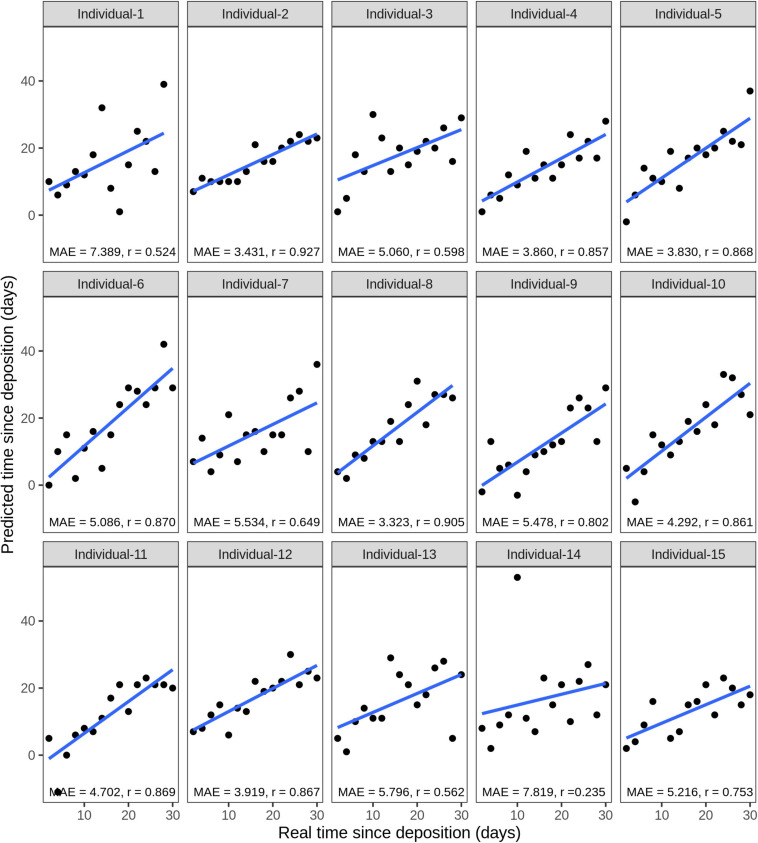
Individual-specific model performance for the prediction of the time since deposition of saliva stains, using data of the short-term (up to 1 month) stored saliva stains based on four bacterial species from 15 individuals. Data from sample duplicates 2 were used for model testing, while data from sample duplicates 1 were used for model building. The mean absolute error (MAE) measures the discrepancies between the real and the predicted time since deposition values. The correlation between real and predicted values is indicated with *r*.

Considering the testing stains of all 15 individuals, the average correlation between the true and predicted time since deposition was *r* = 0.742, while the average MAE was 5 days (16.7% of the analyzed time frame of 1 month). The model for individual 8 presented the lowest MAE of 3.3 days with a correlation between real and predicted values of *r* = 0.905 (Figure 8). The model for individual 14 presented the highest MAE of 7.8 days with a correlation between real and predicted values of *r* = 0.235 (Figure 8). The real and predicted times since deposition for each individual are summarized in Supplementary Table 5. We further investigated errors in the time since deposition prediction in the individual-specific approach (Supplementary Figure 4). There was no clear pattern relating to certain individuals or time points with lower errors. The time since deposition of eleven stains (5.0%) was correctly predicted with zero days error. In the rest of the predictions, there was a similar distribution in the stains in which time since deposition was underestimated (113 stains, 50.9%) or overestimated (98 stains, 44.1%). In 81.5% of the cases (181 stains), the error of the predicted time since deposition fell to within 1 week (up to ±7 days error). From those, more than half of the samples fell within 3 days (±3 days error). More precisely, 16.7% (37 stains) were predicted with ±1 day; 17% (38 stains) with ±2 days; and 9% (20 stains) with ±3 days error.

Additionally, we estimated the time since deposition of short-term dried saliva stains from the six individuals that we collected and exposed to indoor conditions 7 months after the first collection time point. Considering the tested stains of all 6 individuals, the average MAE was 8.8 days (29.3% of the analyzed time frame of 1 month). The one-variable predictor model for individual 1 presented the lowest MAE of 3.9 days. The four-variable predictor model for individual 2 presented the highest MAE of 16.9 days. The MAE values of all the individual-specific models of one, two, three, and four species as predictors for time since deposition can be found in Supplementary Table 7.

## Discussion

Knowledge of the time when a human biological stain was left at a crime scene – also known as time since deposition – can be of great forensic value in assessing the alibis of known suspects, searching for suspects, selecting the stains with the highest informative value for further analysis, helping in missing person cases when the time they went missing is unknown, and in estimating PMI in scenes involving a corpse, or parts thereof. In this study, we evaluated for the first time a microbial DNA-based approach to estimating the time since deposition of dried saliva stains exposed to indoor conditions.

First, we identified the most abundant and most frequent bacterial species in human saliva samples from two publicly available 16S rRNA gene sequencing data sets (Wu et al., 2016; McDonald et al., 2018). On the one hand, we looked for abundant species to ensure their detection in forensic-type saliva samples, which are often as small as a few microliters (μL) in volume. On the other hand, we also looked for frequent species, meaning that they are more likely to be present in the general population; hence, in the great majority of saliva stains found at crime scenes. For our final choice, we focused on species that are both abundant and frequent species at the same time since taxa “exclusive” to an individual often account for a significant percentage of an individual’s microbiome profile (Costello et al., 2009; Nasidze et al., 2009; Zaura et al., 2009; Lazarevic et al., 2010; Huse et al., 2012; Li et al., 2013, 2014; Hall et al., 2017). The top 15 bacterial species identified as both most abundant and most frequent in our samples belonged to genera previously reported to be predominant taxa in saliva and part of the so-called “core” oral microbiome (Costello et al., 2009; Nasidze et al., 2009; Zaura et al., 2009; Lazarevic et al., 2010; Huse et al., 2012; Li et al., 2013, 2014; Hall et al., 2017). Our observations agree with these previous studies, reporting that the saliva microbiome is dominated by just a few taxa, while most of the taxa detected per individual are rare.

Second, we analyzed the microbiome profiles of dried saliva stains exposed to our laboratory environment long-term (up to 1 year) and focused our time-dependent analysis on the previously selected 15 most abundant and frequent bacterial species. Based on differential abundance analysis, we identified four species, the abundance of which significantly changed over time since deposition: *F. periodonticum, H. parainfluenzae, V. dispar*, and *V. parvula.* It is noted that three of the species are obligate anaerobes (*F. periodonticum, V. dispar*, and *V. parvula*), while the fourth one is a facultative anaerobe (*H. parainfluenzae*). Theoretically, obligate anaerobes might be depleted upon exposure to an oxygen-rich environment such as our laboratory, as a previous study also reported this for *Veillonella* genus (Salzmann et al., 2019). We hypothesize that the selected bacterial species co-aggregate *ex vivo* with other saliva microbes forming biofilms and having access to nutrients and molecules for survival and protection. The removal of oxygen by aerobic and facultative anaerobes could create “pockets” of anoxia that support the growth of obligate anaerobes, in a process similar to what happens in human dental plaque both *in vivo* (Schaechter, 2009) and *ex vivo* (Diaz et al., 2002). Obligate anaerobic organisms can also metabolize oxygen and produce protective enzymes in response to oxidative stress (Marquis, 1995; Jabłońska and Tawfik, 2019). Additionally, the bacterial *ex vivo* co-aggregates could be advantageous for the more efficient utilization of nutrients and molecules found in saliva, as previously reported (Bradshaw et al., 1994; Kuramitsu and Ellen, 2000; Periasamy and Kolenbrander, 2009). The method we employed reported only relative abundance changes of the four species over time, though different scenarios could explain the direction of these changes. For example, the increase in the selected reference log ratio balance from 7 to 365 days since deposition could be explained by one of the following five scenarios of absolute abundance changes: (i) the numerator’s taxa increased on average; (ii) the denominator’s taxa decreased on average; (iii) a combination of the previous two happened; (iv) both the numerator’s and denominator’s taxa increased, but the numerator’s taxa increased more compared to the denominator’s taxa; and (v) the numerator’s and denominator’s taxa both decreased, but the denominator’s taxa decreased more compared to the numerator’s taxa.

In line with our findings, other published studies also indicated time-dependent microbiome changes in the dried saliva samples exposed to indoor conditions. Though not the main aim of their study, Salzmann et al., 2019 analyzed the microbial communities in both the fresh and dried saliva samples exposed to their laboratory environment for both 5 and 9 months. Via differential abundance analysis using DESeq2 (Love et al., 2014) they showed that four facultative and obligate anaerobic bacteria were significantly depleted upon exposure to indoor conditions: *Actinomyces, Staphylococcus, Veillonella*, and an unclassified genus from the *Leptotrichiaceae* family. However, no definitive conclusions could be drawn due to the small sample size in the study (*n* = 4, two fresh and two dried saliva stains). Moreover, the analysis employed for the differential abundance testing (DESeq2; Love et al., 2014) was originally developed for RNA-Seq data and requires further development for general use on microbiome data (Weiss et al., 2017). While the authors did not report their results at the species level, looking at the genus level two of the four bacterial species we selected as time-dependent markers belong to the genus *Veillonella*, and one of the 15 species identified as most abundant and most frequent belong to the *Actinomyces* genus and another to the *Leptotrichiaceae* family.

We are aware of the limitation that only two individuals were studied in the differential abundance analysis of the long-term dried saliva stains. This was mainly due to the technical and financial restrictions of our NGS analysis, but we consider it sufficient for a proof of principle study. In the future, analysis of more individuals for targeted time periods will add to these results and perhaps reveal additional promising biomarkers. Our study also only analyzed the four most promising bacterial species via a targeted analysis. However, based on the promising follow-up results, future work could focus on the analysis of all the top 15 most abundant and frequent species as identified from the publically available adult human saliva 16S gene NGS data sets. The other eleven bacterial species might potentially similarly participate in an *ex vivo* microbial consortium (Kolenbrander, 2011). Hence, potential time-dependent changes in their abundance could serve as powerful additional estimators of the time since deposition of saliva stains.

Based on the four differentially abundant and frequent bacterial species, we developed a 4-plex qPCR assay to test the forensic applicability in dried saliva stains exposed to indoor short-term conditions of up to 1 month. A prerequisite for applying such a qPCR assay in a stain would be to confirm its body fluid source being saliva. For this, it is possible to apply another microbiome-based approach for the conclusive identification of saliva stains, as we recently showed in a previous study (Díez López et al., 2019). The four species targeted with the 4-plex qPCR assay were detected in the fresh saliva (t0) of all 15 analyzed individuals, confirming that they are abundant and frequent enough for forensic use. Interestingly, the qPCR-reported cell counts of our four targeted bacterial species increased with time since deposition for the majority of the analyzed individuals. However, high inter-individual differences were observed in the variation explained by time in the species abundance. Nevertheless, there was no clear relationship between a higher explained variation and initial species abundance (t0). We are not very surprised by this, as this variation between individuals could be explained by bacterial interactions. For example, other bacteria taxa present in the sample might have interacted with our targeted species in different ways (i.e., mutualism, syntrophism, commensalism, proto-cooperation, antagonism, competition, parasitism, and predation). The presence and abundance of certain nutrients and molecules in the saliva at the time since deposition could also favor or impair some of these interactions. Finally, while qPCR is a well-established and suitable method for this study, it is also possible to transfer the protocol to newer, more sensitive methods, such as digital droplet PCR, in the future.

For estimating the time since deposition of our saliva stains, we first built a generalized prediction model. By this, we attempted to estimate the time when an “unknown” test stain was deposited based on a previously established model. However, the high inter-individual variation in our data set limited the possibility of implementing such a model. Though time-dependent changes in the four targeted bacteria occurred in the short-term dried saliva stains (up to 1 month) from all the 15 analyzed individuals, the magnitude and evolution through time of those changes were very specific to each individual. As a result, the estimation of the time since deposition of an individual’s stains based on a model trained with stains from other individuals was not feasible with our data set. To further explore the possibility of a generalized model, future studies might employ a much bigger sample size; not only regarding the number of individuals but also regarding the tested environmental conditions. This could enable a better understanding of whether a broad range of inter-individual and different environmental effects can be captured during model building. These effects, together with the bacterial-based time-dependent information, might result in a generalized model being applicable to unknown stains that originate from random individuals in the population, exposed to different environmental conditions.

Based on the limitation of applying a generalized model in our data set, we decided to build individual-specific models to predict the time since deposition of these short-term dried saliva stains. For each individual, we employed the first sample duplicate set for model training and the second for model testing. With this, we aimed to mimic forensic investigations with our data set, where the estimation of the time since deposition of one or various stains is possible based on a model built from a reference set of dried saliva stains from the same individual exposed to adequate storage conditions and time frames (e.g., indoor storage conditions for a particular period of time; Figure 1E). We acknowledge that different factors might affect the model building and accuracy; particularly, the environment the stain is exposed to; i.e., temperature, relative humidity, ambient light, availability of nutrients, and molecules, the template bacterial community present in the stain at the time of deposition (which seems to be affected by individual characteristics), and the time since deposition itself. All these factors are expected to influence the time-dependent changes in the bacterial biomarkers, which could be accounted for by an individualized solution as we do in our data set in more dedicated future studies. The reported MAE values in these individual-specific models further highlighted the observed inter-individual differences, ranging from 3.3 to 7.8 days (average of 5 days). In contrast to RNA-based studies, we did not observe increased prediction errors with increased storage times. There is only one published RNA-based study on the time since deposition estimation of dried saliva stains we can compare our results with (Asaghiar and Williams, 2020). That study reported a slightly lower MAE value (3.5 *vs.* 5 days); however, the sample size was much larger in our study (222 samples from 15 different individuals *vs.* 5 samples). A different study analyzing dried blood stains reported a time estimation error of 2–4 weeks for stains exposed less than 6 months, which we improved in our study for dried saliva stains (Fu and Allen, 2019).

This study observed an increase in the MAE values in the short-term dried saliva stains collected 7 months later, ranging from 3.9 to 16.9 days (average of 8.8 days) compared to a range of 3.3 to 7.8 days (average of 5 days) from the first sample collection. In the same way that different environmental factors can affect the PMI of human cadavers (Belk et al., 2018; Dash and Das, 2020), the time since deposition estimation of dried body fluid stains could be affected by variations in the exposure conditions. We hypothesize that one factor affecting our predictions might be the season, since the first round of stains were collected and exposed during spring and the second round during autumn, with the consequent differences in average temperatures (slightly lower in autumn) and daylight duration (8 *vs.*11 h), even in indoor conditions (since stains were placed 4 m away from a window). It could also happen that the *in vivo* abundance of some of the four selected bacterial species varied between the two saliva collection time points, 7 months apart from each other, which could happen due to changes in an individual’s health status or lifestyle habits, affecting the subsequent time-dependent bacterial abundance changes in the prepared stains. However, given the more extensive previous data sets demonstrating time-wise microbiome stability in saliva *in vivo* (Costello et al., 2009; Lazarevic et al., 2010; Stahringer et al., 2012; Zhu et al., 2012), and the absence of available information on potential changes in our volunteers’ health status and lifestyle habits, our preliminary data need to be considered with care and larger data evidence needs to be established in the future.

More dedicated future research might focus on increasing the reliability of the individual-specific prediction models by, for example, increasing the model training sample size. For instance, instead of preparing and collecting dried stains every 2 days, shorter time frames could be analyzed (i.e., daily or every a few hours), which may better reflect the rapid division rates of bacterial cells when the conditions are favorable. A bigger training set would also mean the possibility of investigating more complex prediction models that can capture other time-dependent changes that are less linear. Additionally, DNA-based analysis can be reliable in time-dependent bacterial “growing” patterns but might present limitations in “decaying” patterns since living and dead cells cannot be distinguished. An alternative could be bacterial RNA-based analysis in which only the live bacterial fraction is analyzed or a combined approach of bacterial DNA/RNA analysis. Before our proposed approach is considered for future forensic applications various forensic developmental and implementation criteria will need to be met. For instance, future research should deal with the suitability of the approach under different scenarios, such as sample volume and sample substrate (e.g., cigarette butts, chewing gums, food utensils, and fabrics) as well as environmental factors (e.g., average temperature, percentage of air humidity, and ambient daylight hours).

## Conclusion

To the best of our knowledge, this research is the first to show from a forensic standpoint, how commensal human bacteria absolute abundance changes can be used to estimate the time since deposition of dried saliva stains. We focused on the abundant and frequent commensal bacterial species of the saliva of human adults, aiming for applications in forensic-type saliva stains from the general population. We observed that, though high inter-individual variation was found, the four selected bacterial species present a high and significant correlation between their abundance in saliva stains and the time since deposition of saliva stains. The data set presents an individual-specific solution for estimating stain time since deposition. We hypothesize that this might be forensically feasible when a saliva reference sample is used to produce the prediction model underlying data based on samples stored for different time intervals under specific environmental conditions that resemble those to which the crime scene stain was exposed, such as in cases of indoor crimes, though more dedicated future research is needed to confirm our hypothesis. While we consider 1 month as a forensically realistic time frame between stain deposition at a crime scene and reference sample collection from potential suspects for the majority of forensic cases, shorter or longer time spans could be studied in more detail to analyze the extended potential forensic utility of our approach.

Our proof-of-principle study suggests that like in other forensic applications, the human microbiota has promising future forensic applications for estimating the time since deposition of a saliva stain at a crime scene. In the future, this novel approach may be expanded to other forensically relevant human stains containing microbial DNA. Before such microbiome-based stain timing can be further considered for practical forensic applications, further microbiome research is needed to better understand and model all the factors contributing to the bacterial time-dependent changes. Additionally, various forensic developmental and forensic implementation criteria will need to be met via more dedicated studies in future.

## Data Availability Statement

The processed NGS and qPCR data produced and used in this study to perform our analysis and derive our conclusions are included in Supplementary Data Sheet 1.

## Ethics Statement

The studies involving human participants were reviewed and approved by Medische Ethische Toestsings Commissie Erasmus MC (MEC-2018–1731). The patients/participants provided their written informed consent to participate in this study.

## Author Contributions

AV conceptualized the work. CDL and AV designed the study with contributions by MK. CDL performed all experiments and data analyses. CDL and AV interpreted the data with contributions by MK. MK provided resources. All authors wrote the manuscript and approved its final version.

## Conflict of Interest

The authors declare that the research was conducted in the absence of any commercial or financial relationships that could be construed as a potential conflict of interest.
