# Supplementary material for: Estimating the Time Since Deposition of Saliva Stains With a Targeted Bacterial DNA Approach: A Proof-of-Principle Study
Source: Front Microbiol. 2021 Jun 2;12:647933. doi: 10.3389/fmicb.2021.647933 (PMC8206545; doi:10.3389/fmicb.2021.647933)
Supplement: Supplementary file 1 [file Presentation_1.pdf]

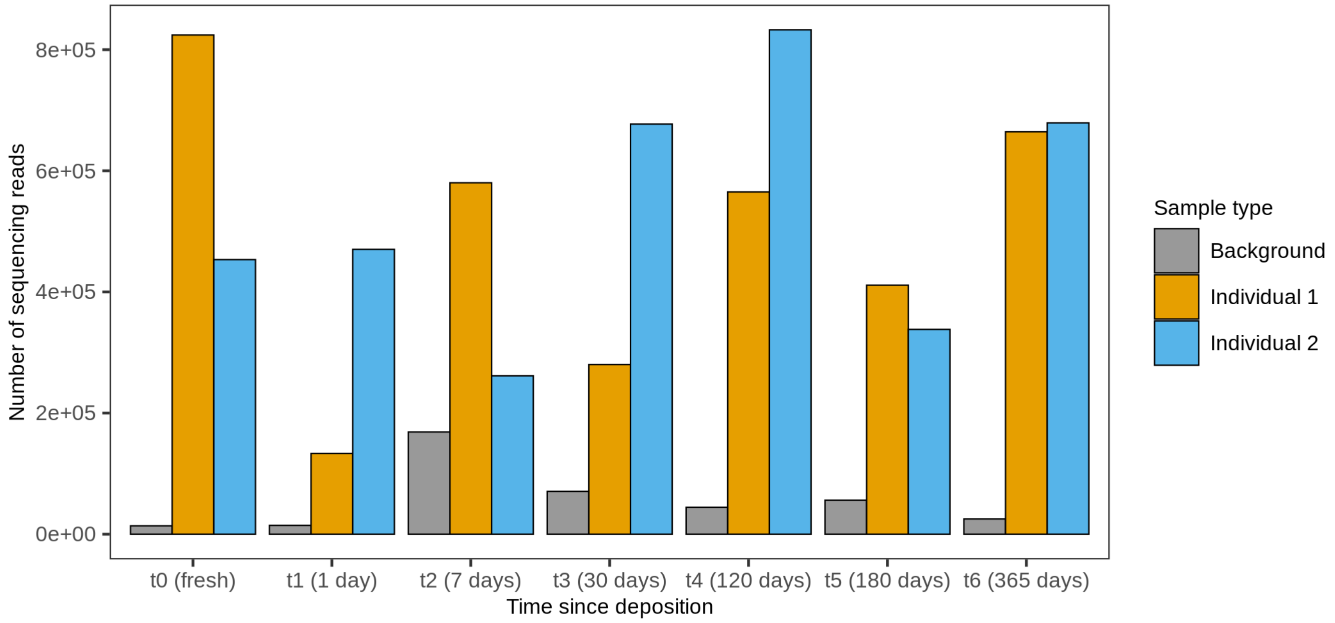

Supplementary Figure 1. Number of 16S rRNA gene (V4-V5 hypervariable region) next-generation sequencing reads obtained from the long-term dried saliva stains which were exposed to indoor conditions up to 1 year. The bar plot includes the background blanks (empty swabs) and the saliva stains produced from the two individuals analysed.

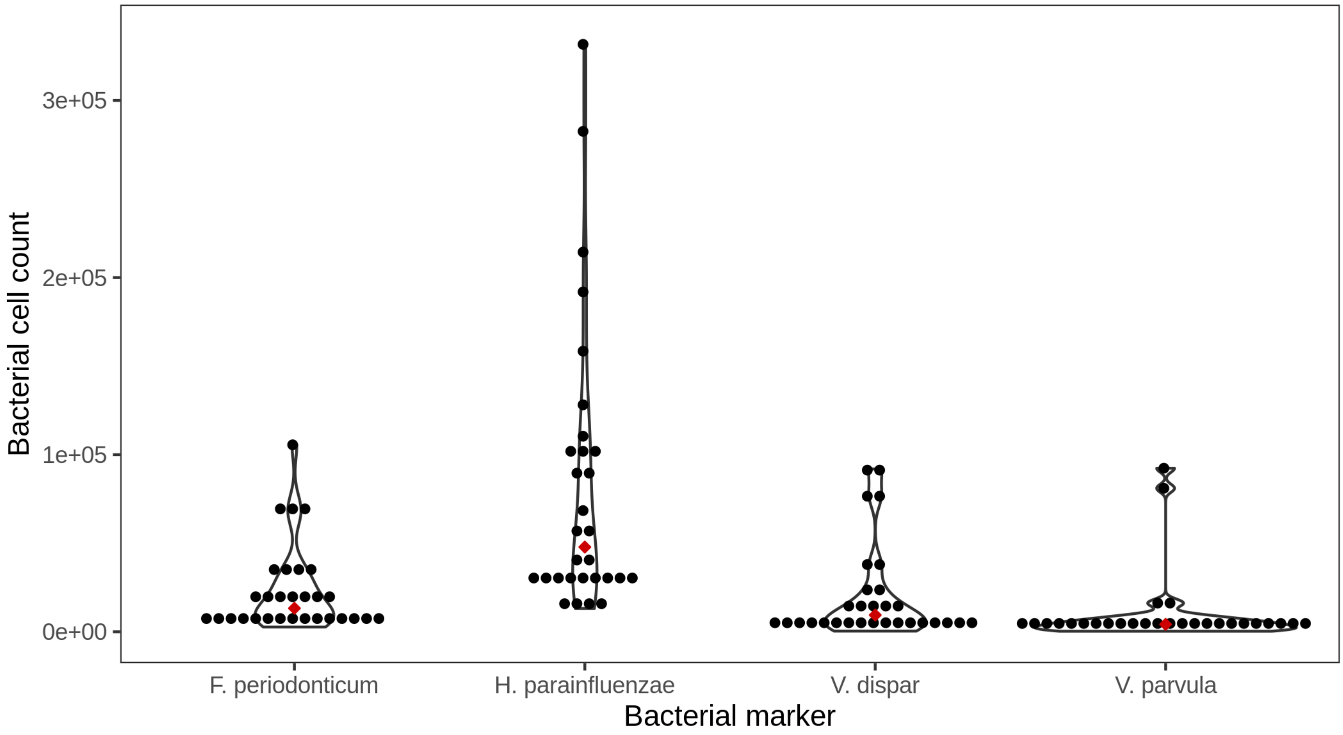

Supplementary Figure 2. Violin plot of the bacterial cell counts for each of the four species in the fresh (t0) short-term dried saliva stains using our developed 4-plex qPCR approach. Each black dot corresponds to a sample duplicate for each of the 15 individuals analysed. Red dots indicate the average cell count for each bacterial marker considering the 15 individuals altogether. The violin plot reveals high inter-individual variation within and between the bacterial markers.

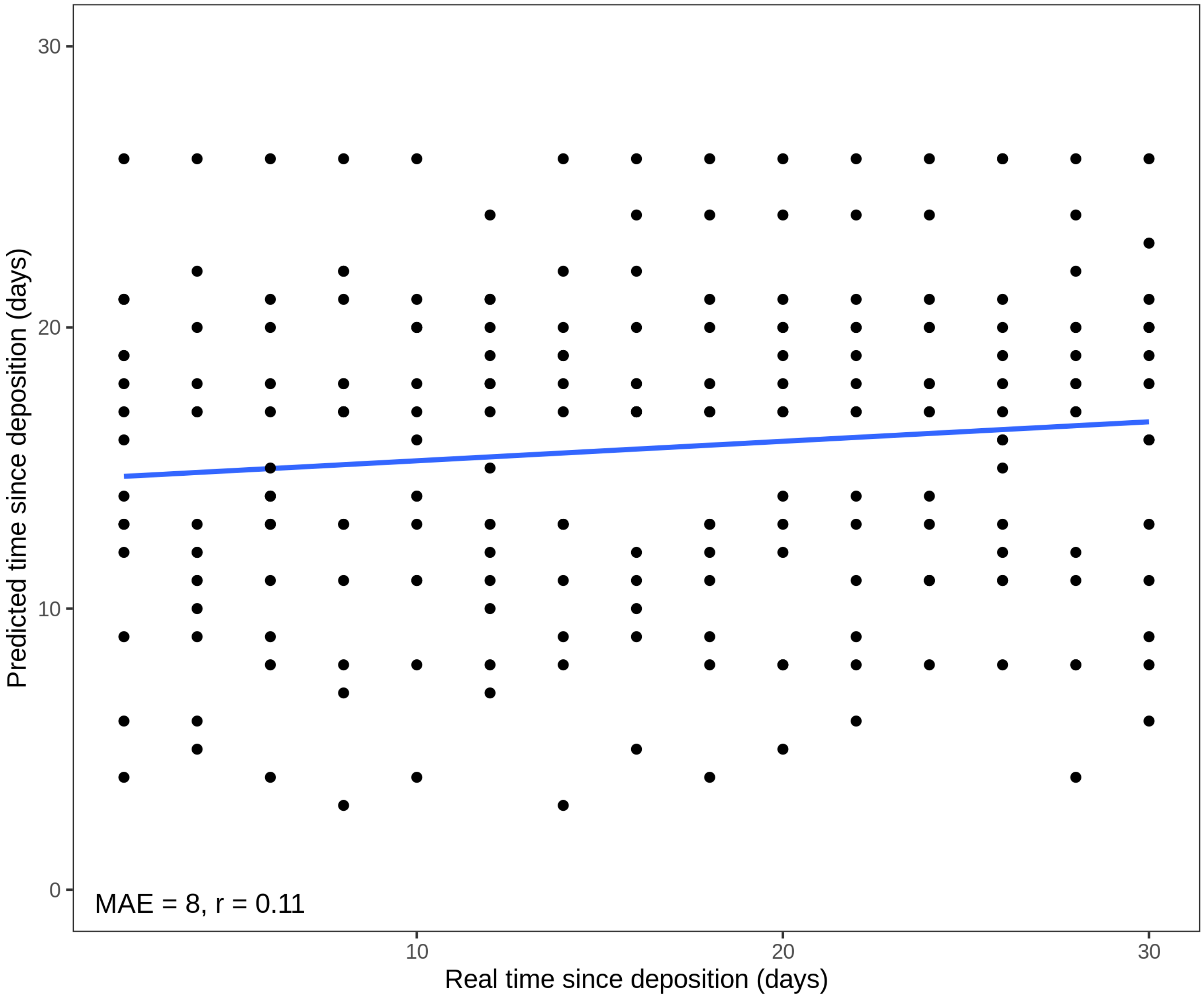

Supplementary Figure 3. Generalized random forest model performance for the prediction of the time since deposition of saliva stains using data of the short-term (up to 1 month) experiment based on four bacterial species from 15 individuals. The mean absolute error (MAE) measures the discrepancies between the real and predicted values of time since deposition. The correlation between real and predicted values is indicated with r.

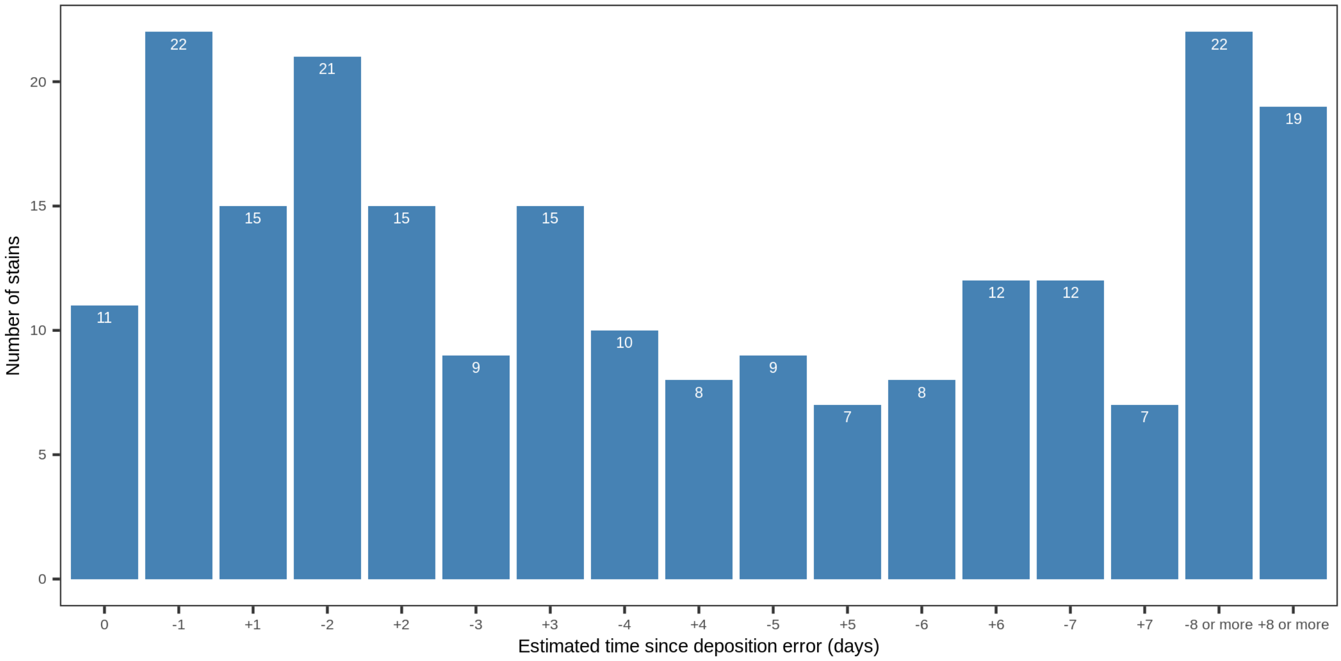

Supplementary Figure 4. Error (in days) from individual-specific stain estimation time since deposition using data of the short-term (up to 1 month) stored saliva stains based on four bacterial species from 15 individuals. Data from sample duplicates 1 were used for model training, while data from sample duplicates 2 were used for model testing. Each bar represents the error in days, from 0 (exact time predicted) up to +/- 8 or more days of error in the predicted time. The height of each bar corresponds to the number of stains that fall within the specified error.
